# Variation in the relationship between birth weight and subsequent obesity by household income

**DOI:** 10.1186/s13561-017-0154-6

**Published:** 2017-05-15

**Authors:** Jonas Minet Kinge

**Affiliations:** 1 Norwegian Institute of Public Health, Pb 4404 Nydalen, 0403 Oslo, Norway; 2 Department of Health Management and Health Economics, University of Oslo, Oslo, Norway

**Keywords:** Birth weight, Obesity, Body mass index, Socioeconomic status, Sibling fixed-effects

## Abstract

There is evidence to suggest that high birth weight increases subsequent BMI. However, little attention has been paid to variations in this impact between population groups. This study investigates the relationship between high birth weight and subsequent obesity, and whether or not this relationship varies by household income. Data was taken from fourteen rounds of the Health Survey for England (between 2000–2014; *N =* 31,043) for children aged 2–16. We regressed obesity in childhood against birth weight, accounting for interactions between birth weight and household income, using sibling-fixed effects models. High birth weight was associated with increased risk of subsequent obesity. This association was significantly more pronounced in children from low-income families, compared with children from high-income families. A 1 kg increase in birth weight increased the probability of obesity by 7% in the lowest income tertile and 4% in the highest income tertile. This suggests that early socioeconomic deprivation compound the effect of high birth weight on obesity.

## Background

The well-known fetal origins hypothesis stresses the importance of fetal development in links between measures of fetal and infant health and later-life health outcomes [1, 2]. In relation to this, it has been suggested that intrauterine growth rate is closely linked to the fetal development of tissues and organs that in postnatal life control eating patterns, physical activity, and metabolism, such as the hypothalamus, pancreatic b-cells, fat tissue, and muscles [3–6]. Hence, a question of interest is whether the prenatal period affects later risk of overweight and obesity. A vast number of studies, including a systematic review and a meta-analysis, have found an association between high birth weight and the risk of overweight in children, adolescents, and adults [7, 8]. The interpretation of these associations may be difficult, however, because many of the studies compared persons who were born to different mothers and brought up in different families. Hence, several researchers have examined the within-family association between birth weight and later body mass index (BMI) in siblings, using sibling-fixed effects designs, and found significant associations [6, 9–11].

While the association between birth weight and subsequent obesity is established, little attention has been paid to variation in this effect between population groups. Such considerations are pertinent given that theory and evidence suggests that parents allocate their investments unequally among their children, and by this reinforce or compensate for initial differences [12]. A growing number of studies also suggests that investment responses vary by family socioeconomic status (SES).

Parental investment responses to a high birth weight may vary by a family’s SES because expectations for children’s BMI, parenting knowledge about diet and exercise, and the availability of resources to respond to childhood obesity. It has been suggested that parental resources may affect (a) preferences for equity in their children’s outcomes, (b) the productivity of investments in obese children, and (c) knowledge about potential ways to compensate for a high BMI, leading some parents to reinforce and others to compensate [13, 14].

**Fig. 1:**
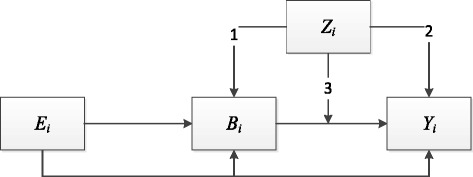
The impact of birth weight on subsequent obesity. E = genetic endowments; B = birth weight; Z = socioeconomic status; Y = obesity; i = indexes individuals

This means that the relationship between birth weight (BW) and later BMI might vary by socioeconomic factors. The framework in Fig. 1 show that household environment, which captures parental behavior for individual *i* (*Z*_*i*_), may affect subsequent obesity (*Y*_*i*_) in three ways. It affects obesity indirectly via its effect on birth weight (*B*_*i*_), which in turn affects subsequent obesity (arrow 1); it affects obesity directly (arrow 2); and, it affects obesity by modifying the relationship between birth weight and obesity (arrow 3).

The aim of the following study is to measure the third effect (arrow 3), i.e., whether or not the relationship between BW and later BMI varies by household environment, which in this study is measured by SES. A small but growing literature has examined whether a family’s SES mitigates the effects of poor child endowments on child outcomes [15–20]. These studies find that poorly endowed children achieve worse outcomes and that the negative impacts of a poor endowment are often larger for children born in disadvantaged families. For example, Almond, Edlund [15] examine the effect of pollution from the Chernobyl disaster on the Swedish cohort that was in utero at the time of the disaster. Those who suffered the greatest radiation exposure were less likely to qualify for high school, and had lower math grades. However, the damage was much larger among those children whose parents were less educated. We have not found any studies that have looked at whether or not the impact of high birth weight on obesity varies by the family SES.

To investigate whether or not the relationship between birth weight and subsequent obesity varies by SES we used data from the Health Survey for England (HSE). The HSE contains measures of children’s anthropometrics, household characteristics (like SES) and other individual characteristics. The survey is representative for English children and includes the BW of children aged 2–16 years. This dataset provides us with a unique opportunity to examine the effects of high BW on obesity by SES.

### Interactions between SES and high BW

There are a number of theories suggesting that health issues may have consequences that are more negative in children from low SES families, compared with high SES families. Currie and Hyson [21] offers three hypotheses predictive of an interactive effect between SES and BW on later life health outcomes. These hypotheses refer to the model of parental investments in children’s human capital. In this model, parents who are assumed to care about their children’s outcomes, maximize their own utility subject to a production function for outcomes and a budget constraint. Although Currie and Hyson [21] discuss the impact of low BW on health and human capital, their discussion has been modified to the case for high BW and later life obesity.

The first hypotheses regarding interactive effects relate to the production function for child health. The idea is that the efficiency with which inputs can be transformed into outcomes may be permanently altered by the fact of the children’s BW. If low-SES children with high BW suffer from a higher incidence of “obesity increasing environmental influences”, than high BW children from high-SES families, this theory predicts that they will have a higher prevalence of obesity.

Currie and Hyson [21] secondly discuss hypotheses that focus on differences in taste between high-SES and low-SES groups. Suppose for example, that BW reflects unmeasured maternal behaviors that continue to affect the outcomes of the child after birth. Such behaviors could be related to exercise and diet as both pre-pregnant body mass index and maternal weight change has an impact on offspring birthweight [22, 23]. A mother that is unwilling to take actions necessary to improve the health of the newborn (e.g. exercise and diet during pregnancy), may also be less likely to make costly investments after the child is born (e.g. help the child with achieving a healthy weight). That is, BW can be influenced by investment in the prenatal period, and this investment could continue after birth. If the propensity to invest in children correlates with social class, then we will observe that high BW has a more pronounced effect on obesity in low-SES children.

Finally, Currie and Hyson [21] discuss hypotheses that focus on the constraints facing parents. For example, the poor face credit constraints, which prevent them from making worthwhile investments in their children. E.g., investments could be in a lifestyle intervention or participation in costly children’s sports. Related to this Conley [24] proposed a theory that resource-allocation decisions vary by social class. When resources are limited, concentrating resources on higher-ability children may be the least risky strategy to ensure success of at least one child. Hence, low SES families may be forced to concentrate limited resources on the ablest child to maximize positive returns for their investments. Conversely, socially advantaged families have more options. They have the means to ensure that high-ability children obtain the minimal level of investments to secure success while directing a higher share of resources toward lower-ability children in an effort to compensate for initial endowment differences. This theory is largely supported by findings from Hsin [14], who used time diaries to investigate maternal time investment in response to birth weight.

The theories and empirical findings above are based on studies of low birth weight and subsequent outcomes. Whether or not it is likely that similar mechanisms apply in terms of high birth weight and subsequent obesity can be explored empirically.

### Data and variables

#### Data source

The analysis was based on data from fourteen rounds (2000–2014) of the *Health Survey for England* (HSE) [25]; 2014 is the most recent year of data available. We excluded 2003 as no information on BW is available that year. The HSE is a repeated cross-sectional survey, which draws a different sample of nationally representative individuals living in England each year. To maximize the sample size we included the children boost samples when available.

All adults (16+) within the household (up to a maximum of 10) are eligible for interview, plus up to two children (0-15). The interviewer randomly selects the children to interview in a household with more than two children. For children aged 0–12, parents answer on behalf of the child, but the child is present.

#### The dependent variable

We used BMI from measured height and weight values measured by the interviewer. One useful feature of the HSE is that the BMI values are not based on self-reported height and weight, which reduces the likelihood of measurement error. We measured obesity as a binary variable taking the value one if a child was obese and zero otherwise. The obesity cut-off values were based on BMI and are age- and gender-specific, defined according to WHO guidelines for children aged 5–19 years [26] and 2–5 years [27]. This means that the definition of obesity varied by age and gender.

#### Birth weight (BW)

BW was recorded for all children under the age of 16. This was done by the interviewer asking the parent or legal guardian about the BW of the children taking part in the survey. As we have relied on parents’ recall of BW, this may be inaccurate. However, prior studies have shown good agreement between maternal recall and medical records of their pregnancy and child’s birth outcomes [28–30]. In fact, Lederman and Paxton [31] stated, “Maternal recall is a satisfactory substitute for clinical data, being consistent with the record, and more complete, yet easier to obtain for clinical studies.”

We treated the BW variable as a continuous variable. However, as the relationship between BW and subsequent obesity might be nonlinear we have repeated the analyses with a categorical BW variable. More details and the results of this analysis is in Appendix 1.

#### Household income

We used household income as our measure of SES. This measure is available for both the boost and the core samples, while parents’ education or occupation is only available for the core sample, if we used these it would reduce our sample size substantially. Following Case, Lubotsky [32], Currie, Shields [33], the main measure of income that we used was current total pretax annual household income, which is provided in 31 bands in the data, ranging from less than £520 to more than £150,000. We took midpoints of these bands. Since the data were collected over a period of 14 years we deflated them (to 2005 prices) using the consumer price index for the UK. Hence, we have a pseudocontinuous measure for total family income, which was then equivalised using McClements household score provided in the HSE to account for household size and composition. Finally we converted the equivalised household income into natural logarithms for use in the analyses.

#### Covariates

We included the following covariates in the regressions: age; sex; interactions between age and sex; ethnicity (white/non-white); Government Office Region (GOR) of residence (nine categories); survey year (fourteen categories). In addition, we included a dummy variable for being the oldest sibling, as birth order has a known effect on health [34]. We also control for maternal age at delivery (five categories <20, 20-30, 30–40, 40+, missing maternal age (2.5% of the children)) as both high and low maternal age is associated with reduced health in the child [35]; and a dummy variable for whether or not the child was born prematurely.

Rather than stratifying by sex, we controlled for it in the analysis. Two recent reviews did not reveal a different association between birth weight and overweight/obesity by sex [7, 8]. In addition, we tried to include interactions between BW and sex in our models below and they were not significant (*p* = 0.23).

## Methods

We modelled childhood obesity for individual *i* as:

1 $$ {Y}_i=\kern0.5em {c}_0\kern0.5em +{c}_1{B}_i\kern0.5em +\kern0.5em {c}_2{Z}_i\kern0.5em +{X}_i\gamma \kern0.5em +\kern0.5em {u}_i $$

where *Y* is a binary measure of obesity for individual *i*; *B* is a measure of BW; Z is a measure of household income; and *X* is a vector of individual, maternal and household characteristics. *u* is an error term and *c* and *γ* are coefficients to be estimated. To allow for the effect of birthweight to vary by income we ran a second model including an interaction between BW and income:

2 $$ {Y}_i={c}_0+{c}_1{B}_i+{c}_2{Z}_i+{c}_3{Z}_i\mathrm{x}{B}_{\mathrm{i}}+{X}_i\gamma +{u}_i $$

were the ZxB is an interaction between household income and BW. If the Eq. [2] has a better fit than Eq. [1] it suggests that the impact of BW varies significantly by household income. We tested this by a likelihood ratio test.

We cannot rule out that omitted variables bias the relationship between BW and obesity. As children are brought up in different families both early life conditions and parental background factors can affect BW and obesity. To mitigate this we ran the following model using sibling-fixed effects specifications:

3 $$ {Y}_{ij} = {c}_0 + {c}_1{B}_{ij} + {X}_{ij}\gamma +{\varepsilon}_j + {u}_{ij} $$

4 $$ {Y}_{i j} = {c}_0 + {c}_1{B}_{i j} + {c}_3{Z}_j x{B}_{i j} + {X}_i\gamma +{\varepsilon}_j + {u}_{i j} $$

where *ij* denotes individual *i* in family *j*, and *ε* represents a family fixed effect. This means that we compared only siblings within each family, and X is a vector of control variables that are not shared between siblings. These are: birth order; maternal age at delivery; whether or not the child was born prematurely; and, the child’s age interacted with gender. Household income does not vary across siblings, which means that we cannot include household income in itself in the models. However, the interaction between household income and BW (ZxB) might still be unique for each sibling and is included in Eq. 4.

Our outcome of interest is a limited dependent variable and our primary models are linear probability models (LPM) with heteroscedasticity robust standards errors. This will yield the best least squares approximation of the true conditional expectation function and we can interpret the coefficients as marginal effects. There are two reasons for choosing LPM over, e.g., logit or probit models. Firstly, the interpretation of interactions in non-linear models is less clear as the coefficients are multiplicative [36]. Secondly, non-linear fixed effects models has been shown to provide biased results [37], [Greene W, Han C, Schmidt P. The bias of the fixed effects estimator in nonlinear models. Unpublished Manuscript, Stern School of Business, NYU. 2002;29]. However, we reran part of our regressions with logit models and calculated marginal effects for interactions according to Ai and Norton [36]. The conclusions did not change.

We apply survey weights reported in the HSE to each observation. The individual survey weights were generated separately for adults and children. For children (aged 0 to 15), the weights were generated from the household weights and the child selection weights – the selection weights corrected for only including a maximum of two children in a household. The combined household and child selection weights were adjusted to ensure that the weighted age/sex distribution matched that of all children in co-operating households. The survey weights were not used in the regressions with sibling-fixed effects.

It is possible that observations are independent across households, but not within households. We therefore also controlled for clustered sampling within household using unique household identifiers that produced Huber/White/sandwich robust variance estimators that allowed for within-household dependence [38].

## Results

Roughly, two thirds of the full sample was also included in the sibling analyses (Table 1). Although the sociodemographic characteristics were comparable, the obesity prevalence was higher in the full sample (11.2%), than in the sibling sample (10.5%).

**Table 1:** Summary statistics of the estimation samples

	Full	Sibling
Total (N)	31,043	19,460
Male (N)	15,718	9,806
Female (N)	15,325	9,654
Birth weight (mean)	3.34	3.35
Obese (%)	11.2	10.5
Age (mean)	9.0	9.1
Maternal age at birth (mean)	28.9	28.7
Preterm birth (%)	5.0	5.6
LN household income (mean)	9.8	9.8
Survey year (%)		
2000	1.09	1.10
2001	7.70	7.79
2002	16.93	17.80
2004	3.16	3.19
2005	6.13	6.20
2006	0.32	0.37
2007	15.86	15.78
2008	15.41	15.36
2009	8.37	8.24
2010	11.17	10.86
2011	3.29	3.06
2012	3.40	3.53
2013	3.73	3.43
2014	3.46	3.29

**Fig. 2:**
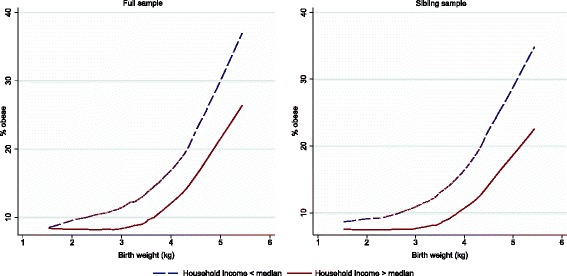
The association between birth weight and obesity in the full sample and the sibling sample. Split into two groups: income above median an income below median

Predicted smoothed values of subsequent obesity by BW are in Fig. 2. The predictions are based on locally weighted regressions, with a bandwidth of 0.8. They show that obesity prevalence increased with BW. This pattern was found both in the children with a household income above and below the median. However, the figures suggest that the obesity prevalence increased relatively more in the children with a household income below median, compared with the children in families with an income above median. The findings in the full sample and in the sibling sample were similar, though the differences by household income appear to be more pronounced in the sibling sample.

Linear BW was associated with increased risk of obesity, while the log of household income was associated with reduced risk of obesity in regressions with covariates (Table 2). The interaction between BW and income was significant and the LR-test suggests a significantly better fit in the model with the interaction. These results were found in the full sample and the sibling sample. The BW-coefficient was significant in the sibling fixed effects specification. In addition, the interactions between income and BW remained significant in the sibling fixed effects models. This suggests that the impact of BW on subsequent obesity varies significantly with household income. The effect of BW was larger in children from low-income families.

**Table 2:** OLS of the effect of birth weight in kilos (continuous) and income on obesity in the full sample and the sibling sample

	No interactions (Eq. 1/Eq. 3)		With interactions (Eq. 2/Eq. 4)	
	*Coef.*	*t*	*Coef.*	*t*
Full sample*				
Birth weight	0.0403	9.81	0.1273	3.04
Log of household income	-0.0220	-8.11	0.0078	0.56
Birth weight X income			-0.0089	-2.13
*LR test of basic model vs. model with interaction*			*p = 0.01*	
Sibling sample*				
Birth weight	0.0353	6.77	0.1503	2.76
Log of household income	-0.0227	-6.51	0.0170	0.94
Birth weight X income			-0.0118	-2.17
*LR test of basic model vs. model with interaction*			*p = 0.01*	
Sibling fixed effects**				
Birth weight	0.0434	5.64	0.1874	2.34
Log of household income	*[equal across siblings]*		*[equal across siblings]*	
Birth weight X income			-0.0148	-1.8
*LR test of basic model vs. model with interaction*			*p < 0.01*	

* Covariates: age; sex; interactions between age and sex; ethnicity; Government Office Region (GOR) of residence; survey year; birth order; maternal age at delivery; and, whether or not the child was born prematurely

** Covariates: birth order; maternal age at delivery; whether or not the child was born prematurely; and, the child’s age interacted with gender

When we stratified the children into tertiles by income, we also observe a more pronounced effect of BW on obesity, in the lowest income tertile (Table 3). Similar findings were done in the sibling fixed effects analysis, where the effect of BW, on obesity, was 0.067 in the lowest income tertile while it was 0.036 in the highest income tertile.

**Table 3:** The effect of the birth weight (continuous) on the probability of obesity. Stratified by household income. Results for the full sample and for sibling fixed effects analysis

	Full sample		Sibling fixed effects	
	*Coef.*	*t*	*Coef.*	*t*
All	0.0375	10.58	0.0434	5.64
Low income	0.0521	7.59	0.0670	4.5
Medium income	0.0329	5.28	0.0310	2.37
High income	0.0354	6.52	0.0356	2.92

Covariates: birth order; maternal age at delivery; whether or not the child was born prematurely; and, the child’s age interacted with gender

As shown in Fig. 2, the effect of BW on subsequent obesity might not be linear. Hence, we reran the analysis with a categorical BW variable. The findings, which are presented in Appendix 1, supports the results, presented in Tables 2 and 3 using linear BW. In low-income children, the association between high BW and obesity was significantly more pronounced, compared with high-income children.

## Discussion

The aims of this study were to investigate the relationship between birth weight and subsequent obesity, and whether or not this relationship varies by household income. Our main findings were that of a significant correlation between high birth weight and subsequent obesity, and that the correlation varied significantly by household income. The effect of BW on subsequent obesity was more pronounced in children from low-income households compared with children from high-income households.

We provide evidence to show that children born with high birth weight had significantly higher obesity prevalence, than those of normal birth weight. Variables shared between siblings did not explain our findings, according to the sibling fixed effects models. These results are qualitatively similar to those in other studies, which have also shown that high birth weight is associated with increased risk of subsequent overweight and obesity [7, 8]. Our findings also support earlier studies that have used sibling fixed effects and found a significant impact of birth weight on obesity [6, 11]. However, direct comparisons of the coefficients are difficult as most other studies report their findings in odds ratios, while we reported marginal effects. A few studies have shown an association between birth weight and subsequent obesity using UK data [39, 40]. However, this study is, to our awareness, the first study using UK data and a sibling fixed effects design.

Our main finding is however, that the association between birth weight and subsequent obesity varied significantly by household income; the association was more pronounced in children from lower income households. Children born with high birth weight in low-income households were more likely to be obese than those of normal birth weight in low-income household, and were more likely to be obese than those in higher income households who was born with the same birth weight. This trend was also observed after controlling for individual and household characteristics and in sibling fixed effects models. While a number of studies have investigated the impact of birth weight on subsequent obesity controlling for socioeconomic variables in multivariate analyses, we are not aware of any published studies that have stratified their analyses by socioeconomic status to investigate whether or not the association between birth weight on obesity varies by socioeconomic status.

Our findings are consistent with the theories proposed in the introduction. For example, the theory on strategic investment propose that parental allocation decisions largely are driven by conscious investments strategies of the parents [24]. This results in low-income families concentrating their limited resources on the higher-ability children, to maximize their human capital returns. While high-income parents adopt compensatory strategies because they can afford this, i.e. they devote more resources to the less endowed children, while still ensuring enough resources to their other children to maximize their probability of success.

An alternative explanation might be that low income parents might be more likely to lack the psychological and material resources to handle high need children [14]. Caring for high birth weight children might be more burdensome, not only are they at increased risk for being obese, they are also more likely to suffer from a number of chronic illnesses [41, 42] and have worse academic outcomes [43, 44]. Successful obesity interventions does not only demand a persistent effort from the child, they are also more likely to be successful if the parents are involved [45]. In this case, our results would not be a result of conscious investments, but from immediate responses to the current situation. Although our study cannot separate out one of these alternative explanations, it offers a strong empirical finding: the impact of birth weight on subsequent obesity varies by household income.

There are a number of implications of these findings. The fetal origins literature emphasizes fetal development and its consequences for later life health outcomes [1, 2]. However, this study finds that environmental and social factors may alter the biological effect of high BW on later life obesity. This suggests that the conditions in utero is less important for the development of obesity than the epidemiological literature has suggested as the home environment may—depending on whose home it is—promote or prevent the development of subsequent obesity in those being born with a high birth weight. If this is true, studies that do not account for heterogeneity in the effect of high BW on obesity may, on the one hand, overestimate the effect in lower SES families, but, on the other hand, underestimate the effect for children born into high SES families. Our findings also suggest that socioeconomic inequalities in obesity might be related to conditions in utero.

The present study has limitations. First, our measure of obesity was BMI, which has been criticized, e.g., because it does not incorporate body fat, which is an independent predictor of ill health [46]. In addition, earlier studies have suggested that birth weight is positively associated with both lean body mass and fat in adults [47]. Although we used age and gender specific cut-off values for obesity, caution is necessary when BMI is used as children and adolescents can experience growth in height and weight during brief periods [48]. Second, the children, in the sibling fixed effects models, were members of families with more children and were less obese than were the children in the total study population. Thus, the results of the sibling comparisons may not be fully representative for the total population of English children.

## Conclusion

Our study has shown that, as in previous studies, high BW was associated with increased risk of subsequent obesity. In addition, we have shown that the association between BW and obesity was more pronounced in children from lower income households. High household income buffers the effect of birth weight on subsequent obesity.

## Appendix 1

### Treating birth weight as a categorical variable

In the following BW is grouped into three categories. “Low BW” defined as a BW < 2.5 kg; “normal BW” defined as a BW between 2.5 and 4.5 kg; and, “High BW” defined as BW > 4.5 kg.

When BW was included as a categorical variable we found a significant and positive effect of high BW, compared with normal BW, on obesity (Table 4 in Appendix 1). The LR-tests also suggest that the models that included interactions between BW and income had a significantly better fit. I.e. the effect of high BW on subsequent obesity was higher in children from low income families.

When we stratified the children into tertiles by income we also observe that the effect of BW was more pronounced in the lowest income tertile (Table 5 in Appendix 1). Similar findings were done in the sibling fixed effects analysis.

**Table 4:** OLS of the effect of birth weight (categorical variable) and income on obesity in the full sample and the sibling sample

	No interactions (Eq. 1/Eq. 3)		With interactions (Eq. 2/Eq. 4)	
	*Coef.*	*t*	*Coef.*	*t*
Full sample*				
High birth weight	0.0516	7.81	0.2025	2.35
Low birth weight	-0.0263	-2.26	-0.0743	-0.91
Log of household income	-0.0212	-7.85	-0.0197	-6.72
High birth weight X income			-0.0153	-1.78
Low birth weight X income			0.0051	0.61
*LR test of basic model vs. model with interaction*			*p = 0.06*	
Sibling sample**				
High birth weight	0.0458	5.69	0.2293	2.14
Low birth weight	-0.0222	-1.48	-0.0807	-0.8
Log of household income	-0.0218	-6.26	-0.0199	-5.25
High birth weight X income			-0.0187	-1.75
Low birth weight X income			0.0062	0.6
*LR test of basic model vs. model with interaction* ^a^			*p = 0.05*	

* Covariates: age; sex; interactions between age and sex; ethnicity; Government Office Region (GOR) of residence; survey year; birth order; maternal age at delivery; and, whether or not the child was born prematurely

** Covariates: birth order; maternal age at delivery; whether or not the child was born prematurely; and, the child’s age interacted with gender

**Table 5:** The effect of the birth weight (categorical variable) on the probability of obesity. Stratified by household income. Results for the full sample and for sibling fixed effects analysis

	Full sample		Sibling fixed effects	
	*Coef.*	*t*	*Coef.*	*t*
All				
High birth weight	0.0525	9.58	0.0358	3.63
Low birth weight	-0.0127	-1.02	-0.0316	-1.39
Low income				
High birth weight	0.0709	6.03	0.0556	2.7
Low birth weight	-0.0246	-1.17	-0.0203	-0.56
Medium income				
High birth weight	0.0507	5.33	0.0411	2.4
Low birth weight	-0.0159	-0.69	0.0027	0.06
High income				
High birth weight	0.0454	5.78	0.0177	1.23
Low birth weight	-0.0051	-0.24	-0.0750	-1.94

Covariates: birth order; maternal age at delivery; whether or not the child was born prematurely; and, the child’s age interacted with gender

## Abbreviations

BMI: Body mass index
BW: Birth weight
HSE: Health survey for England
LPM: Linear probability models
SES: Socioeconomic status
